# Loss of the *Drosophila* branched-chain α-ketoacid dehydrogenase complex results in neuronal dysfunction

**DOI:** 10.1242/dmm.044750

**Published:** 2020-08-27

**Authors:** Hui-Ying Tsai, Shih-Cheng Wu, Jian-Chiuan Li, Yu-Min Chen, Chih-Chiang Chan, Chun-Hong Chen

**Affiliations:** 1 Institute of Infectious Diseases and Vaccinology, National Health Research Institutes, Zhunan, Miaoli 35053, Taiwan; 2 Institute of Molecular and Cellular Biology, National Taiwan University, Taipei 10090, Taiwan; 3 National Mosquito-Borne Diseases Control Research Center, National Health Research Institutes, Zhunan, Miaoli 35053, Taiwan; 4 Graduate Institute of physiology, National Taiwan University College of Medicine, Taipei 10051, Taiwan

**Keywords:** MSUD, Neuronal apoptosis, Oxidative stress, *Drosophila*

## Abstract

Maple syrup urine disease (MSUD) is an inherited error in the metabolism of branched-chain amino acids (BCAAs) caused by a severe deficiency of the branched-chain α-ketoacid dehydrogenase (BCKDH) complex, which ultimately leads to neurological disorders. The limited therapies, including protein-restricted diets and liver transplants, are not as effective as they could be for the treatment of MSUD due to the current lack of molecular insights into the disease pathogenesis. To address this issue, we developed a *Drosophila* model of MSUD by knocking out the *dDBT* gene, an ortholog of the human gene encoding the dihydrolipoamide branched chain transacylase (DBT) subunit of BCKDH. The homozygous *dDBT* mutant larvae recapitulate an array of MSUD phenotypes, including aberrant BCAA accumulation, developmental defects, poor mobile behavior and disrupted L-glutamate homeostasis. Moreover, the *dDBT* mutation causes neuronal apoptosis during the developmental progression of larval brains. The genetic and functional evidence generated by *in vivo* depletion of *dDBT* expression in the eye indicates severe impairment of retinal rhabdomeres. Further, the *dDBT* mutant shows elevated oxidative stress and higher lipid peroxidation accumulation in the larval brain. Therefore, we conclude from *in vivo* evidence that the loss of *dDBT* results in oxidative brain damage that may lead to neuronal cell death and contribute to aspects of MSUD pathology. Importantly, when the *dDBT* mutants were administrated with Metformin, the aberrances in BCAA levels and motor behavior were ameliorated. This intriguing outcome strongly merits the use of the *dDBT* mutant as a platform for developing MSUD therapies.

This article has an associated First Person interview with the joint first authors of the paper.

## Abstract (precis)

**Summary:** Loss of BCKDH activity in *Drosophila* recapitulates the neurological symptoms of patients with maple syrup urine disease. Metformin administration was found to alleviate developmental defects and aberrant behavior in the BCKDH mutant.

## INTRODUCTION

Maple syrup urine disease (MSUD) is a rare autosomal recessive inherited disease associated with disruption of the normal activity of the branched-chain α-ketoacid dehydrogenase (BCKDH) complex, which is a multi-enzyme macromolecule with four catalytic components (E1α, E1β, E2 and E3) in humans. The loss of activity results in insufficient breakdown and aberrant accumulation of branched-chain amino acids (BCAAs), including leucine, isoleucine and valine. The clinical phenotypes of MSUD are based on the severity of the response to the residual BCKDH enzyme activity and the gene locus affected, including classic MSUD cases, which demonstrate only 0–2% residual BCKDH activity, intermediate MSUD cases that have 2–8% activity, and intermittent MSUD cases that have 8–15% residual BCKDH activity. Worldwide, classic (50–75%) and intermediate (20%) MSUD diagnoses account for the majority of cases (Chuang et al., 2006).

There are currently two therapeutic strategies for treating MSUD: dietary restriction and liver transplantation (Khanna et al., 2006). Of these options, a BCAA-free diet is the most commonly used option, with patients avoiding consumption of food rich in BCAAs (Blackburn et al., 2017). Dietary compliance can be difficult, however, as this restriction can lead to a deficiency in essential amino acids. Furthermore, evidence indicates that a protein-restricted diet can lead to a decline in crucial antioxidant abilities in animal models (Sitta et al., 2014), and for patients with MSUD, this may occur after long-term treatment with dietary restriction. Liver transplantation is theoretically the preferred treatment, as it leads to the cessation of all symptoms (Khanna et al., 2006). However, this therapy is limited by high financial costs, limited organ availability and the risks associated with surgery. An alternative approach to treating MSUD is therefore necessary. Developing alternative strategies requires an improved understanding of the fundamental mechanisms of the disease, as well as a pipeline for testing novel therapeutics.

Several model organisms are currently used to investigate MSUD, including Hereford calves (Harper et al., 1986), mice (Homanics et al., 2006) and zebrafish (Roberts, 2012). In 1986, for example, 2-day-old Hereford calves affected with MSUD were used to study central nervous system (CNS) disorders. Diffuse severe status spongiosus of white matter in the cerebellum was found by ultrastructural microscopic examination, suggestive of myelin edema (Harper et al., 1986).

More recently, in 2006, a murine model was generated by utilizing embryonic stem cell technologies to knock out a functional E2 subunit gene, which encodes an ortholog of human dihydrolipoyl transacylase (DBT). However, this classic MSUD model could not be used to study potential MSUD treatments as the mice died within a few days of birth. Instead, an intermediate model was created, which used transgenic technology to express human E2 complementary DNA (cDNA) in the knockout background. BCKDH activity reached 5–6% and was sufficient to allow for survival, but was insufficient to normalize circulating BCAA levels (Homanics et al., 2006). The majority of patients with MSUD exhibit classic cases with detrimental effects seen in the clinic, including a variety of developmental delays, neurological impairments, and even neurodegeneration starting at the neonatal period through to adulthood. Utilization of therapeutic strategies is highly associated with the residual BCKDH activity level; therefore, there is an established need for an efficient model system to study the etiology correlation to subsequent MSUD effects.

In 2012, a new zebrafish model of MSUD was established via mutation of the E2 component. Zebrafish mutant larvae showed abnormal swimming behavior, reductions in the level of glutamate in the brain and nervous system developmental problems (Friedrich et al., 2012). Each of the existing animal models offers different advantages for researchers, and potential treatments for MSUD have emerged from experiments conducted using these models. However, any such therapeutic evidence has been limited by a lack of translation potential to humans, most likely due to the complexity of the underlying pathogenesis of MSUD, which is caused by a number of pathogenic factors such as neurotransmitters (Muelly et al., 2013), metabolic signaling (Biswas et al., 2019) and oxidative stress (Sitta et al., 2014). *Drosophila* with genetic similarities have commonly been used to model human disease (Pandey and Nichols, 2011; Ugur et al., 2016) and can be a powerful method to survey disorders via deciphering underlying mechanisms (Wu et al., 2017). The *Drosophila CG5599* (hereafter referred to as *dDBT*) gene encodes an ortholog of human DBT (the E2 subunit of BCKDH), as based on its acetyltransferase activity and lipoic acid-binding activity. However, this model system is not yet in widespread use for MSUD.

In this study, we generated a *Drosophila dDBT* (BCKDH, E2 component)-deficient mutant by using the CRISPR/Cas9 system, and subsequently used the mutant flies to investigate the physiological effects of BCKDH deficiency. Our data show BCAA accumulation and development defects, as evidenced by poor eclosion rates and smaller body sizes in *dDBT* homozygous mutants. Our results further show that the loss of *dDBT* function leads to a lower glutamate level and impairment of crawling behavior in the larval stage, as well as causes neuronal apoptosis in the brain throughout the larval and pupal stages. In support of observations on the neurological damage caused by the loss of *dDBT*, we found that the heterozygous *dDBT* mutant survives to adulthood, albeit with poor subsequent survival, and that deficits in neuronal function are evidenced by vacuolar lesions in the CNS. Furthermore, we found that the induction of brain oxidative stress correlates with neuronal damage in the absence of *dDBT* activity. Lastly, Metformin treatment was found to improve the abnormal behavior of the *dDBT* mutant. In conclusion, the *Drosophila dDBT* mutant represents a critical defect in neurological physiology, which is similar to the symptoms of a human with MSUD, suggesting that loss of BCKDH function in *Drosophila* recapitulates the critical characteristics of MSUD.

## RESULTS

### Genetic depletion of *dDBT* causes increased BCAA levels in *Drosophila*

**Fig. 1.**
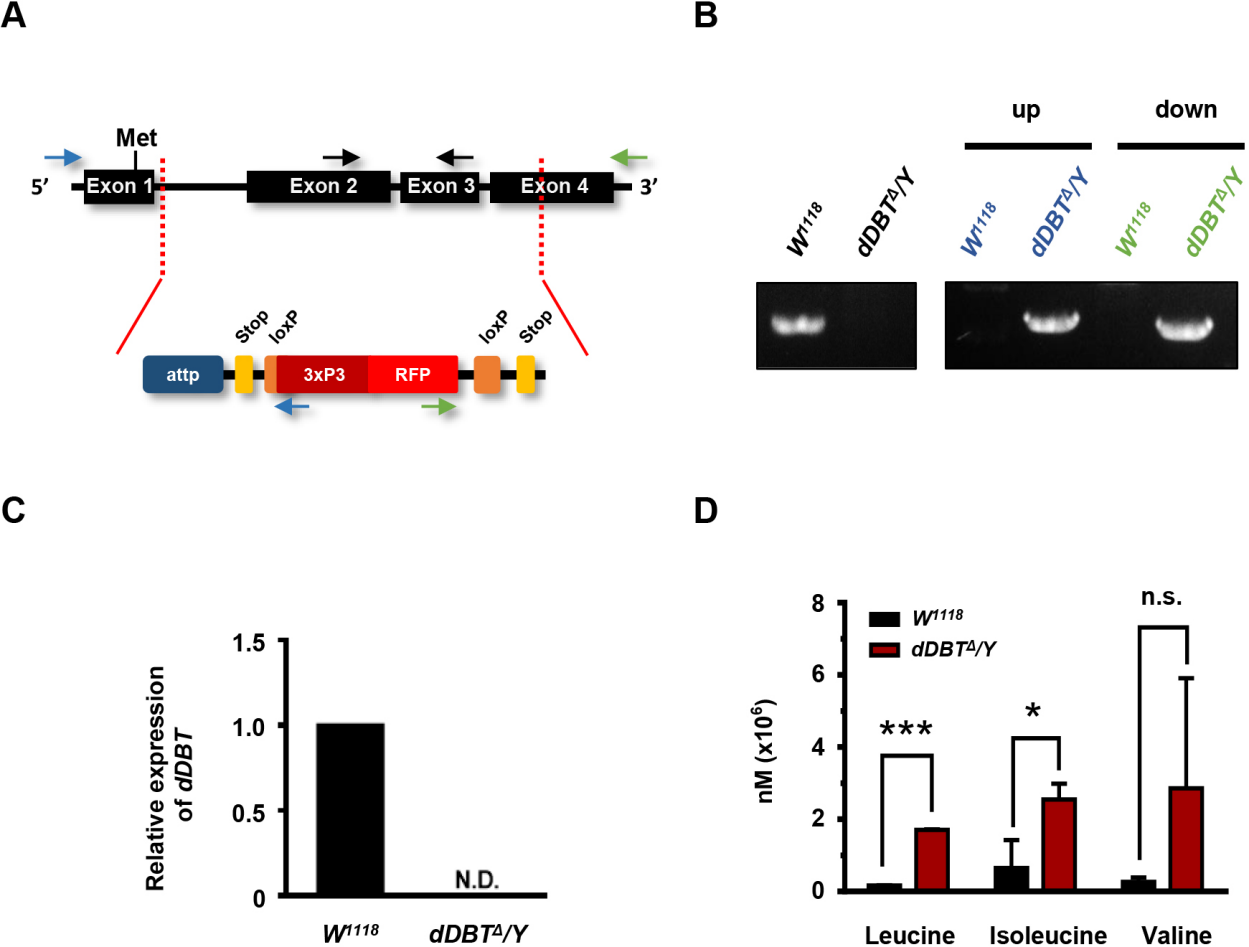
**Increased BCAA levels in the *Drosophila dDBT* mutant generated by the CRISPR/Cas9 system.** (A) Schematic representation of *dDBT* deletion via CRISPR/Cas9 knock-in cassette. Met, methionine. (B) Genotyping of the *dDBT* mutant by genomic PCR analysis with specific primers labeled in black, blue and green. (C) RT-qPCR analysis of *dDBT* mRNA expression. (D) LC-MS analysis of BCAA amounts in larval body fluid (*n*=25, in each group/experiment). All biological samples were harvested and used from *W^1118^* or *dDBT^Δ^/Y* larvae*.* Error bars indicate s.d. *P<0.05, ***P<0.001; N.D., not determined; n.s., not significant (unpaired Student's *t*-test).

To establish a *Drosophila* model of MSUD to study disease pathogenesis and develop potential treatments, we first sought to generate BCKDH knockout flies using CRISPR/Cas9-mediated genome editing utilizing homology-dependent repair (HDR), consisting of two guide RNAs and a double-stranded DNA plasmid donor to delete the *dDBT* gene. The excision began near the start codon, and the majority of the coding sequence was deleted by knocking in a cassette containing attPX, two STOP codons and 3xP3-RFP. As a selection marker, 3xP3-RFP was used to facilitate genetic screening (Fig. 1A). To confirm the CRISPR/Cas9-generated *dDBT* mutant, we determined the gene loci and transcriptional expression of *dDBT* in the CRISPR-generated mutants compared to wild-type *W^1118^* controls. Our data from the genomic PCR revealed that the *dDBT* gene had been knocked out in the mutant (Fig. 1B), and analysis of quantitative reverse transcription PCR (RT-qPCR) showed that *dDBT* mRNA transcripts were no longer present (Fig. 1C). A deficiency in BCKDH activity leads to abnormal BCAA accumulation, which has long been recognized as a pivotal toxic event leading to MSUD (Blackburn et al., 2017). To examine whether BCAA levels changed in the *dDBT* mutant, we quantified BCAA levels via liquid chromatography–mass spectrometry (LC-MS), and found a significant accumulation of BCAAs, such as increased leucine and isoleucine, in the *dDBT*-deficient mutant compared to wild-type controls (Fig. 1D). Together, these results suggest that *dDBT* has a crucial role in modulating the homeostasis of BCAA catabolism in *Drosophila*.

### Loss of *dDBT* disrupts developmental progression

**Fig. 2.**
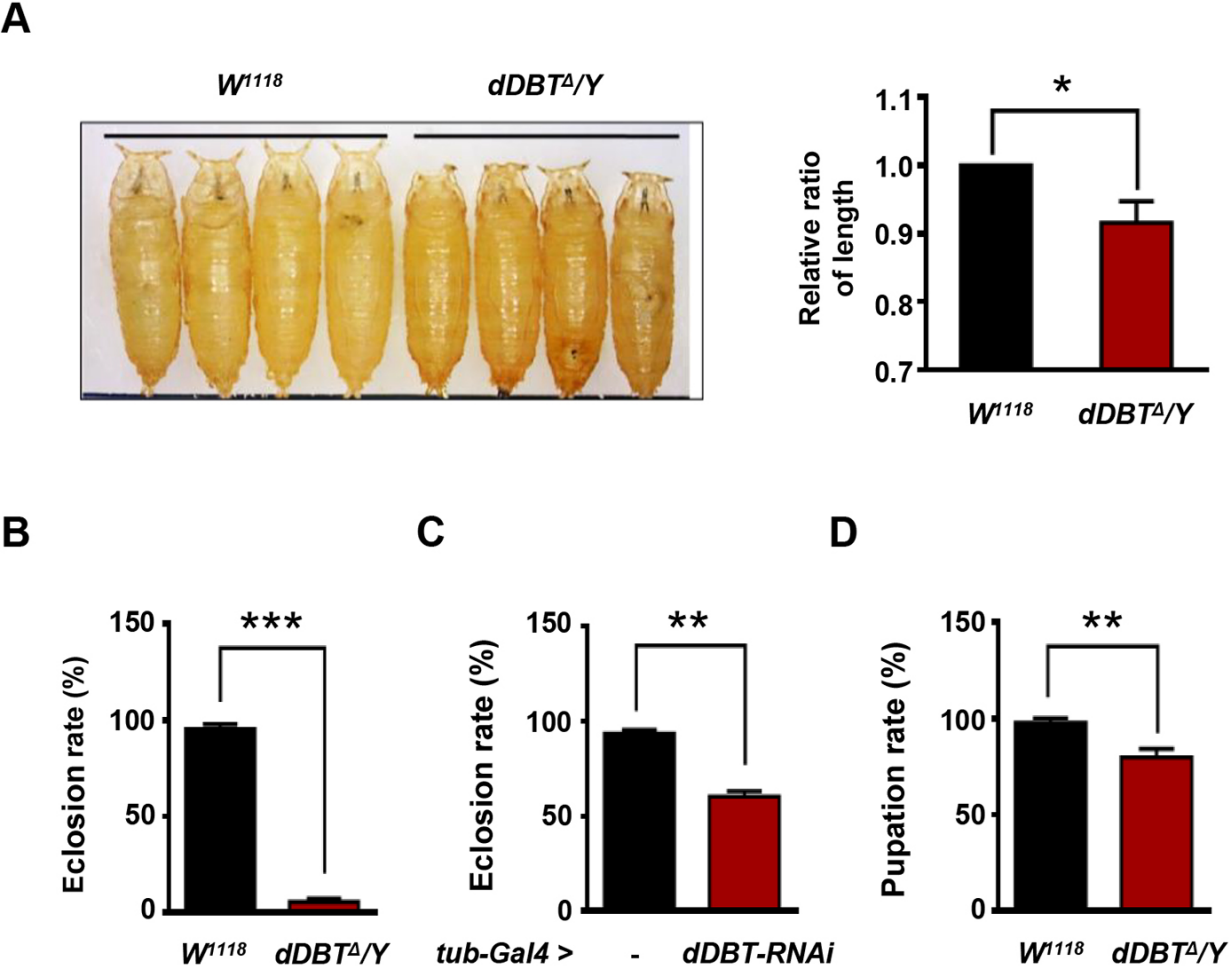
**Loss of *dDBT* elicits developmental defects in *Drosophila*.** (A) Analysis of pupal length (*n*=20, each group/experiment) from *dDBT^Δ^/Y* compared to *W^1118^*. (B,C) Analysis of eclosion rate (*n*=35, each group/experiment) from strains of *dDBT^Δ^/Y* compared to *W^1118^* (B) or from strains of *tub-Gal4>dDBT-RNAi* compared to *tub-Gal4* alone (C). (D) Analysis of pupation rate (*n*=35, each group/experiment) from flies of *dDBT^Δ^/Y* compared to *W^1118^*. Three biological repeats were conducted; error bars indicate s.d. *P<0.05, **P<0.01, ***P<0.001 (unpaired Student's *t*-test).

Branched-chain amino acid catabolism is essential for normal physiological function (Lynch and Adams, 2014). When untreated, responses to aberrant BCAA elevation include developmental delays and neurological symptoms, which are common observations in patients with MSUD. We investigated whether *dDBT* mutants were able to recapitulate critical symptoms related to MSUD with aberrant levels of BCAAs. In our observations of developmental progression, we found significant differences between controls and mutant lines in terms of pupal size and eclosion rate. The majority of the *dDBT* mutant pupae demonstrated shorter body lengths (Fig. 2A) and could not eclose, with eclosion rates of ∼7.6% (Fig. 2B). Those that did eclose died within a few hours. To avoid interference from the genetic background, we alternatively depleted *dDBT* function via a genetic Gal4-UAS system (Brand and Perrimon, 1993). Supporting our observations of BCKDH function in developmental progression, ubiquitously decreasing *dDBT* expression (*tub-Gal4>UAS-dDBT-RNAi*) also resulted in a lower eclosion rate than in controls (*tub-Gal4* alone) (Fig. 2C). Further, we observed a reduced pupation rate for *dDBT* mutants compared to wild types (Fig. 2D), suggesting that developmental deficits can occur during *dDBT* mutant larval stages. Together, these results suggest that BCAA accumulation is toxic to immature forms of *D. melanogaster*, a similar phenotype to that observed in humans with the classic form of MSUD.

### The *Drosophila dDBT* mutant shows MSUD-like symptoms

**Fig. 3.**
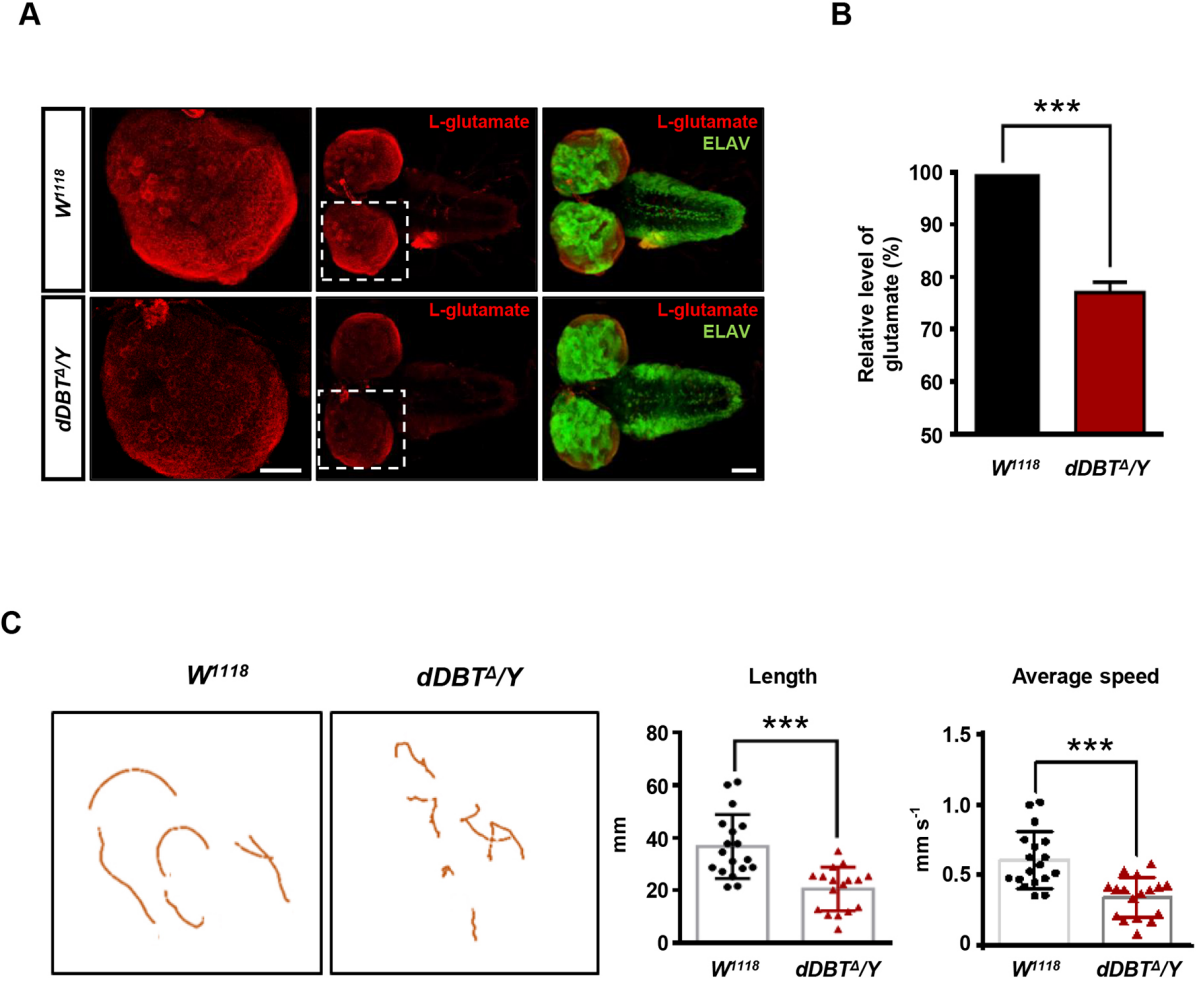
**Decreased L-glutamate levels and poor motility in the *Drosophila dDBT* mutant.** (A) Confocal images of the L-glutamate levels in larval brains stained with anti-ELAV (green) or anti-L-glutamate (red) antibody. (B) Quantification of the L-glutamate level in larval brains using a fluorometric L-glutamate assay kit. Brains used for examination of L-glutamate were from *W^1118^* or *dDBT^Δ^/Y* larvae (*n*=100, each group/experiment). (C) Analysis of the crawling behavior of *W^1118^* or *dDBT^Δ^/Y* larvae (*n*=6, each group/experiment) recorded by video tape. Representative images are shown (ImageJ). Three biological repeats were conducted; error bars indicate s.d. ***P<0.001 (unpaired Student's *t*-test). Scale bars: 100 μm.

Chronic neuropsychiatric sequelae is a major complication of MSUD, with neurotransmitter deficiencies, such as glutamate, thought to be responsible for the neurotoxicity (Muelly et al., 2013). To further characterize the effects of the loss of *dDBT* in *Drosophila*, we investigated whether glutamate homeostasis was affected in the *dDBT* mutant. Reduced levels of L-glutamate were found in the brains of *dDBT* mutants (Fig. 3A), ∼25% less than in the wild-type brains (Fig. 3B).

In addition, movement disorders can be manifested by MSUD (Carecchio et al., 2011), as well as various metabolic diseases (Gouider-Khouja et al., 2010). Reduced levels of neurotransmitters correlated with altered motor behaviors in mouse models of MSUD (Zinnanti et al., 2009). Thus, we hypothesized that phenotypic expression, such as motor behavior, would be impaired in the *dDBT* mutant. Crawling behavior was examined, and the results showed significant reductions in travel length and average speed. We also observed differences in crawling patterns, with mutant larvae tending to have uncoordinated paths and fewer turns (Fig. 3C). Our data show that the *Drosophila dDBT* mutant recapitulates the major symptoms of MSUD.

### Neuronal apoptosis is induced by *dDBT* deficiency

**Fig. 4.**
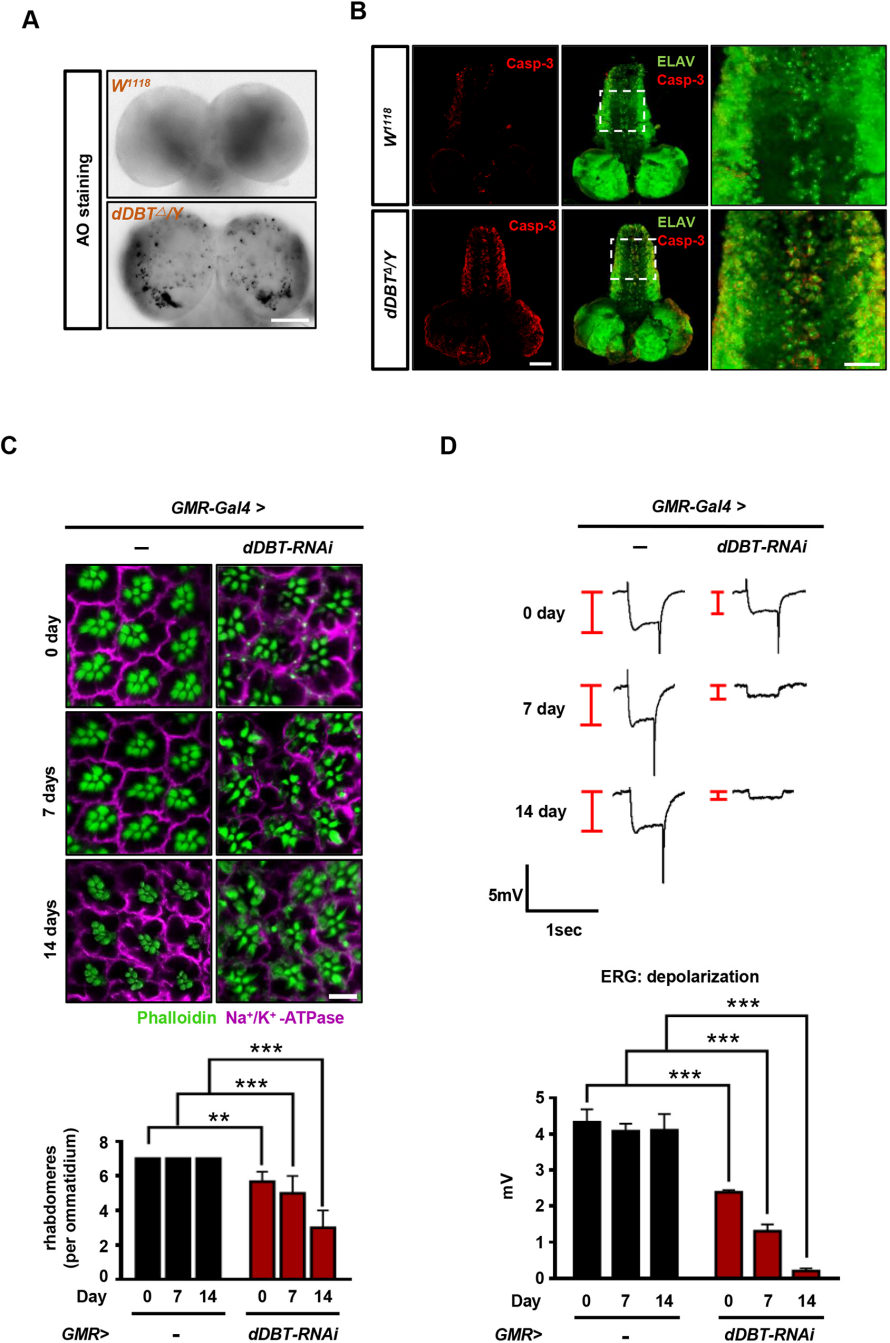
**Loss of *dDBT* activity triggers neuronal cell death in *Drosophila*.** (A,B) Apoptosis assay of larval brains from *W^1118^* or *dDBT* mutants using Acridine Orange (AO) dye (A), or anti-Caspase-3 immunostaining (B). Images of whole-mount larval brains were captured by fluorescence confocal microscopy using a 20× objective. (C) Immunostaining of *Drosophila* photoreceptors after exposure to constant light stimulation for 7 or 14 days (top). Rhabdomeres were stained by phalloidin (green) and the neuronal membrane was stained by Na^+^/K^+^-ATPase (magenta). *GMR-Gal4>UAS-dDBT* RNAi or *GMR* alone flies (*n*=15, each group/experiment) were used for the quantification of the number of rhabdomeres (bottom). (D) Neuronal function as measured by ERG (top). Eyes from *GMR-Gal4>UAS-dDBT* RNAi or *GMR-Gal4* flies (*n*=10 each group/experiment) were used for the quantification of depolarization amplitudes (bottom). Three biological repeats were conducted; error bars indicate s.d. **P<0.01, ***P<0.001 (unpaired Student's *t*-test). Scale bars: 100 μm (A,B), 5 μm (C).

The nature of MSUD in humans suggests a highly complex neuronal disruption. A growing body of evidence suggests that injection of large amounts of BCAAs induces neuronal cell death *in vitro* and *in vivo* (Jouvet et al., 2000; Vilela et al., 2017). However, the effects of neuronal cell death on the neuropathology of MSUD remain obscure. Our data show that *dDBT*-deficient mutants were mostly arrested at the pupal stage (Fig. 2B). We therefore hypothesized that cell death would appear in the pupal brain of *BCKDH*-deficient mutants. By determining cell death via immunostaining with apoptosis markers, our results showed severe neuronal apoptosis in the pupal brain of the *BCKDH* mutants (Fig. S1). Because the severity of neuronal death is strongly correlated with the temporal and spatial toxicity of BCAA (Jouvet et al., 2000), we hypothesized that the toxic effects might occur in larval progression prior to the pupal stage, supported by the locomotion deficits observed in mutant larvae (Fig. 3C). To test this, we examined the results of Acridine Orange (AO) chemical staining or anti-Caspase-3 immunostaining, which showed a large number of apoptotic neuronal cells in the larval CNS of *dDBT* mutants (Fig. 4A,B). This suggests that the loss of *dDBT* function induces neuronal death in the homozygous mutants. Homozygous *dDBT* mutants did not survive to adulthood, but heterozygotes did (data not shown). Therefore, we further evaluated the temporal and spatial effects of *dDBT* function on CNS damage *in vivo.* Our data revealed that vacuolar lesions were present at significant levels in adult brains and shortened the lifespan of the heterozygous *dDBT* mutant compared to that of the wild type (Fig. S2), suggesting that the expression and activity of *dDBT* plays an important role in influencing neuronal death. To further show this, we performed an examination of the neuronal function of *Drosophila* compound eyes, as the *Drosophila* photoreceptor neurons have proven to be a favorable system for studying neuronal cell death (Jackson et al., 1998). When observing morphological changes in photoreceptors, we found a significant decrease in the number of rhabdomeres in the *dDBT* knockdown lines (*GMR-Gal4>UAS-dDBT-RNAi*) in comparison to the control (*GMR-Gal4* alone) in the absence or presence of light, which accelerates the neuronal damage process (Fig. 4C), as the photoreceptors are light sensitive. Moreover, electroretinogram (ERG) data showed a reduction in depolarization and a loss of on-transients in the knockdown line (Fig. 4D), with the trend in agreement with retinal morphology. This clearly shows that the loss of *dDBT* causes neuronal damage or apoptosis, suggesting that neuronal death might be a pivotal event in the neuropathology of MSUD.

### *dDBT* loss triggers a systemic immune response and oxidative damage in the larval brain

**Fig. 5.**
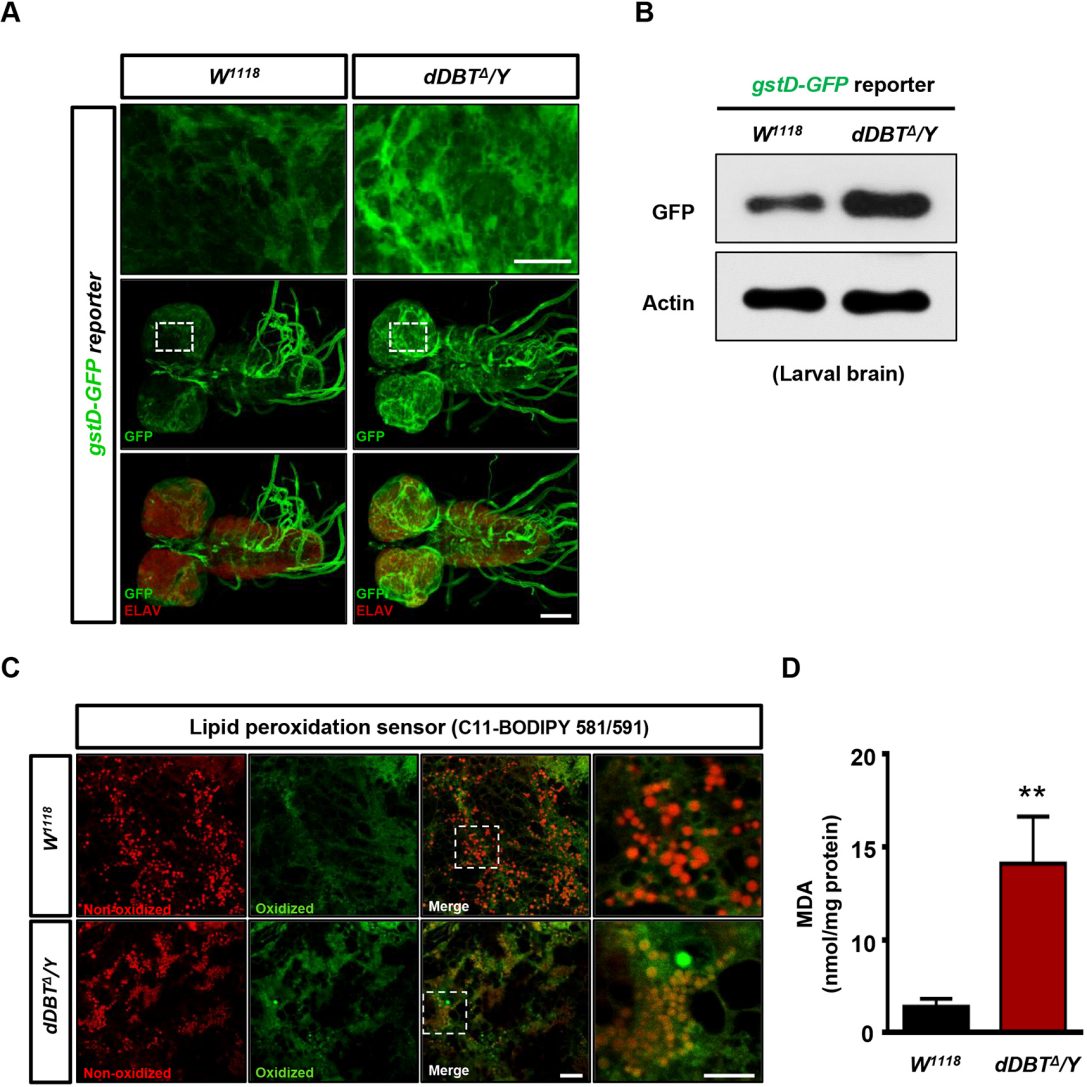
**Loss of *dDBT* activity induces oxidative damage in the larval brain.** (A,B) Confocal images of larval brains (A) and western blot analysis of lysates from whole larval brains (B) from *x/y; gstD-GFP/+* or *dDBT^Δ^/y; gstD-GFP/+* reporter lines*.* The whole-mount brains were co-immunostained using anti-ELAV (red) and anti-GFP (green) or immunoblotted with anti-GFP or anti-Actin antibody. Images were captured by fluorescence confocal microscopy using a 20× objective. (C) Confocal images of lipid peroxidation in the central brain regions of *W^1118^* or *dDBT* larvae mutants. Images were captured by fluorescence confocal microscopy using a 60× oil objective. (D) Quantitative analysis of lipid peroxidation in whole brains from *W^1118^* or *dDBT* larvae mutants (*n*=150, each group/experiment). Three biological repeats were conducted; error bars indicate s.d. **P<0.01 (unpaired Student's *t*-test). MDA, malondialdehyde. Scale bars: 100 μm (A), 10 μm (C).

Recent evidence of higher levels of inflammatory and oxidative biomarkers has been found in the plasma of individuals with MSUD (Mescka et al., 2013; Scaini et al., 2018; Sitta et al., 2014), indicating that these markers might be risk factors for neuropathology in MSUD. However, it is unclear whether inflammation and oxidative stress directly correlate with neuropathological damage in MSUD patients. This question requires *in vivo* study; however, the risks from unbalanced essential amino acids and neurotransmitters to patients with MSUD is great. The *Drosophila* antimicrobial peptide (AMP) immune response shares an evolutionarily conserved mechanism with the inflammatory induction of human cytokines. By examining AMP expression via determining mRNA expression, we found that depleting *dDBT* led to the induction of an AMP response in the brain and fat body, a major immune-responsive organ of *Drosophila* (Fig. S3). Our data of higher humoral immune response in the context of the deficiency of BCKDH activity coordinate with clinical findings (Scaini et al., 2018). Next, we examined whether oxidative stress in the brain is affected by the loss of *dDBT.* We generated *dDBT* mutants carrying a reactive oxygen species (ROS) stress-responsive reporter (*dDBT^Δ^/Y;* *gstD-GFP/+*), to monitor cellular oxidative stress in the brain. Our results showed that ROS stress is strongly elevated in the larval brain of *dDBT* mutants compared with wild-type flies (Fig. 5A,B). Given that ROS stress-induced lipid peroxidation plays a crucial role in cell death, including apoptosis (Gaschler and Stockwell, 2017), we sought to examine whether lipid peroxidation accumulation responds to a deficiency of *dDBT*. Our results revealed that lipid peroxidation accumulates in greater abundance in the brains of the *dDBT* mutants compared to wild-type brains (Fig. 5C,D), and that elevated ROS stress has a detrimental effect in brain damage. This suggests that *dDBT* deficit-induced oxidative damage might be involved in triggering neuronal cell death in the brain.

### Metformin improves developmental defects and crawling behavior caused by *dDBT* deficiency

**Fig. 6.**
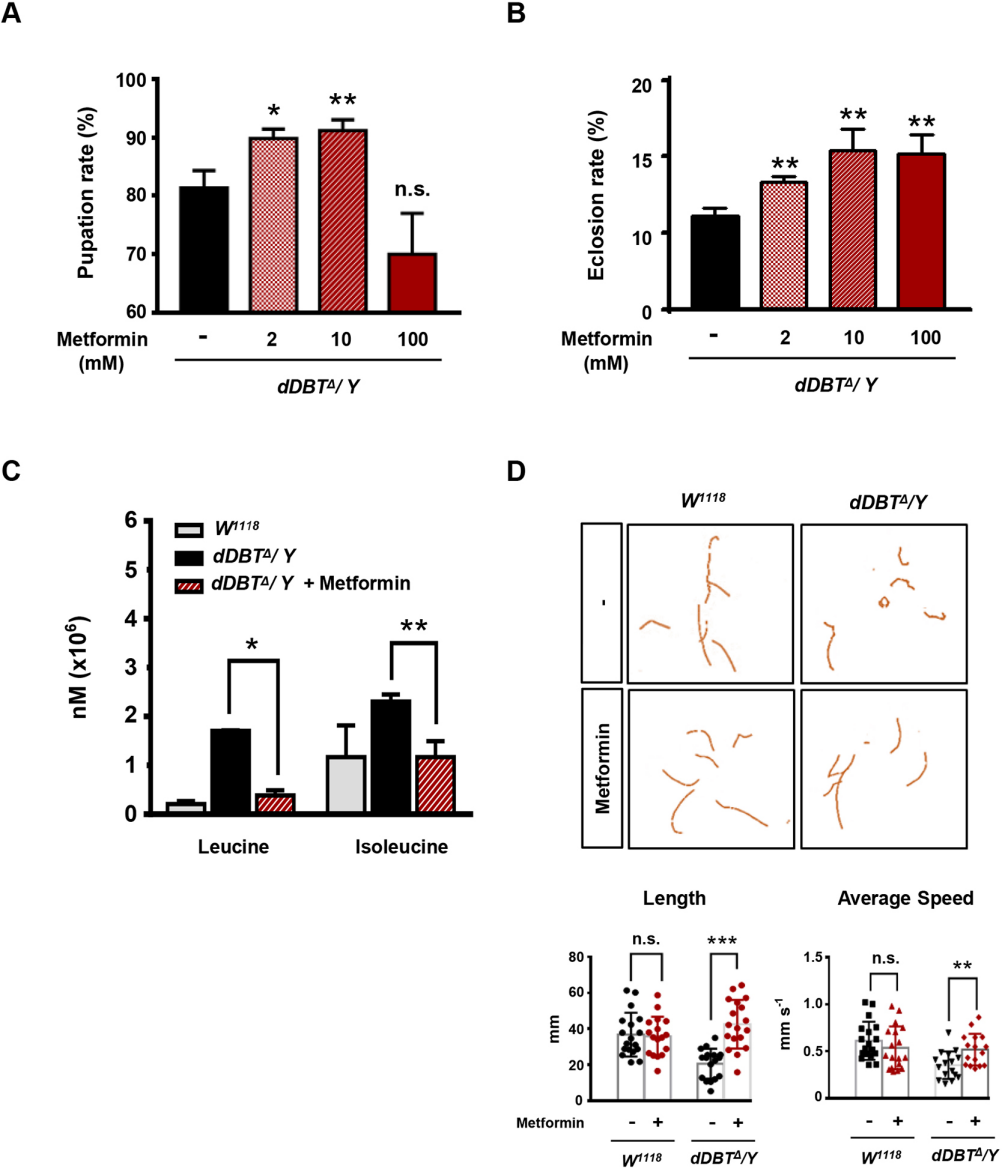
**Metformin administration improves fitness in *dDBT* mutant larvae.** (A,B) Analysis of pupation (A) and eclosion (B) rates from *dDBT^Δ^/Y* larvae orally administrated with or without the indicated concentrations of Metformin (2, 10 or 100 mM). (C,D) LC-MS analysis of BCAA amounts in larval body fluid (*n*=25, each group/experiment) (C) and analysis of crawling behavior (*n*=6, each group/experiment) (D) from *W^1118^* or *dDBT^Δ^/Y* larvae with or without 10 mM Metformin treatment. Three biological repeats were conducted; error bars indicate s.d. *P<0.05, **P<0.01, ***P<0.001; n.s., not significant (unpaired Student's *t*-test)).

Current evidence indicates that Metformin, a widely prescribed antidiabetic drug, lowers BCAAs (Rivera et al., 2020) and keto acids, such as ketoisocaproic acid (KIC) (Sonnet et al., 2016). We therefore hypothesized that Metformin could be beneficial in treating MSUD by offering protection against the detrimental effects provoked by high BCAA levels observed as a result of *dDBT* deficiency. Following administration of various dosage of Metformin, our results showed that both pupation and eclosion rates were significantly improved in *dDBT* mutants in response to Metformin treatment (Fig. 6A,B). To test whether the improvement by Metformin correlated with the amelioration of aberrant BCAA accumulation, the *dDBT* mutant was provided orally with 10 mM Metformin. Metformin administration was found to significantly ameliorate the aberrant accumulations of both leucine and isoleucine levels (Fig. 6C). Accordingly, the poor crawling behavior caused by a loss of *dDBT* was improved in response to the provision of Metformin (Fig. 6D), suggesting that Metformin administration may offer a potential beneficial treatment for ameliorating the symptoms of MUSD.

## DISCUSSION

At present, the lack of effective medications is a major concern in MSUD therapy, as symptoms are still present in individuals lowering BCAA levels through diet (Strauss et al., 2006), and liver transplantation has numerous potential complications (Blackburn et al., 2017; Strauss et al., 2010). To further therapeutic interventions, research on the neuropathological effects noted in MSUD is warranted. We are the first to identify the function of the *Drosophila dDBT* gene and determine its involvement in the regulation of BCAA catabolism. Through knocking out *dDBT* with the CRISPR/Cas9 system, our data from the *Drosophila dDBT* mutant present evidence related to the neuropathological characteristics of human MSUD. This could lead to establishment of a new animal model of MSUD in the *Drosophila* system, which would improve our understanding of the etiology of the disease and assist with the development of new therapeutic options.

In this study, the *dDBT* mutant recapitulates numerous deficiencies seen in MSUD, such as poor motor behavior, premature death and lower neurotransmitter availability, as reflected by glutamate levels in the brain. A growing body of evidence suggests that the aberrant accumulation of BCAAs, or their downstream metabolites, is a potential origin of MSUD. In the present study, higher leucine and isoleucine levels were found in the *dDBT* mutant than in wild type, implying that BCKDH activity is evolutionarily important for BCAA homeostasis in an organism. In clinical observations, MSUD patients also present long-term neurocognitive deficits, which cannot be avoided by dietary interventions (Vogel et al., 2014). Likewise, all psychiatric sequelae could not be eradicated by liver transplantation, which could be explained by the persistent deficiency of cerebral BCKDH contributing to disruption of the neurochemical microenvironment (Mazariegos et al., 2012; Muelly et al., 2013; Shellmer et al., 2011). Moreover, brain responses to high concentrations of BCAAs might trigger various deleterious mechanisms caused by the metabolic imbalance (Scaini et al., 2012). Therefore, in addition to symptoms being irreversible, the mechanisms underlying MSUD may involve the temporal and spatial characteristics of BCAAs and interact with other physical effects. Although inflammation and oxidative stress are known to be pivotal factors for neurological or neurodegenerative disease (Amor et al., 2010; Emerit et al., 2004), clear *in vivo* evidence of MSUD that can be attributed to these factors is lacking. In this study, we show that the loss of BCKHD activity resulting from *dDBT* deficit induced neuronal apoptosis (Fig. 4) and facilitated oxidative stress-induced damage (Fig. 5) in the brain. Importantly, our data showing apoptotic brain damage in the mutant are supported by histological observations of brain injury in the intermediate MSUD model of an E2-deleted heterozygous mouse (Zinnanti et al., 2009), and in tissue from human patients. In addition, a mouse model administrated a high BCAA pool subcutaneously showed memory impairments that could be prevented through the co-injection of an antioxidant, supporting the theory that ROS stress might play a role in brain damage caused by higher levels of BCAAs. Interestingly, studies in patients with MSUD indicate that there is no clear association between BCAA metabolites and markers of ROS stress (Sitta et al., 2014), indicating that various factors are involved in the modulation of MSUD.

Cellular-based screening systems are largely unable to adequately model complex human disease processes that involve multiple cell types across many different organ systems; these can only be emulated by utilizing model organisms (Hellerstein, 2008a,b). The *Drosophila* system offers many advantages, including a short life cycle, genetic manipulability and ease of handling (Jennings, 2011; Pandey and Nichols, 2011), which conveniently allow modeling of complex traits that are relevant to human disease and commonly manipulate for large-scale drug screening (Willoughby et al., 2013). In extension to the applications of the *Drosophila dDBT* mutant beyond studying MSUD etiology, this model could also be used as a practicable platform for drug screening for MSUD. Metformin has been reported to not only decrease circulating BCAA at physiological levels (Rivera et al., 2020), but also BCAA metabolites, such as KIC, in serum and muscle from the mouse model of intermediate MSUD, and in fibroblast cells from MSUD patients (Sonnet et al., 2016). However, the functional rescue of motor behavior and developmental defect in animal models, such as zebrafish and mouse models, of MSUD exposed to Metformin remains ambiguous. The *Drosophila dDBT* deficiency model showed that comparable outcomes of aberrant BCAA accumulation could be halted, and developmental defects and poor motor behavior improved, following Metformin administration. Based on observations in functional rescues with toxic alleviation during the developmental period, our results support the hypothesis that Metformin could offer a potential MSUD treatment opportunity (Fig. 6), as currently there is no effective drug invention for MSUD. Intriguingly, our study demonstrates that the *Drosophila dDBT* mutant provides a useful model of MSUD for studying the underlying pathophysiological events and performing pharmacological evaluations by conveniently examining changes in developmental growth and motor behavior. To achieve translational relevance for our proposed *Drosophila* model as a drug-screening platform, it is next necessary to perform a proof-of-concept test using newly developed compounds. Overall, the *Drosophila dDBT* mutant as a model could offer a number of advantages for the cellular, molecular, pharmacological and genetic analysis of MSUD.

## MATERIALS AND METHODS

### Fly stocks

The *dDBT* mutant used in this study was generated using the CRISPR/Cas9 system (Ran et al., 2013). *tub-Gal4* (BDSC #5138), *GMR-Gal4* (BDSC #84247) drivers and *W^1118^* wild type were obtained from the Bloomington *Drosophila* Stock Center. UAS-*dDBT-RNAi* lines (VDRC #v106456) were obtained from the Vienna *Drosophila* Resource Center. According to the standard protocol, all flies were reared on a standard medium and housed at 25°C.

### Developmental analysis

This study used male mutants (*dDBT^Δ^/Y*) and *dDBT*-depleted mutants (*tub>UAS-dDBT-RNAi*) of the same age. For comparisons of pupae size, total pupal length was measured before eclosion and the difference presented as a relative ratio. To determine eclosion rate, first-instar larvae were picked and reared in density-controlled vials. The number of eclosed pupae was then counted manually. The pupation rate was calculated as the ratio of pupae to the original number of larvae. The eclosion rate was calculated as the ratio of the number of adults to the original number of pupae.

### Measurement of circulating BCAA levels

*Drosophila* hemolymph was tested via liquid chromatography-based metabolomics as described previously (Matsushita and Nishimura, 2020). In brief, third-instar larvae were collected, washed with PBS and dried on tissue paper. Hemolymph was then gathered from the larvae by gently pulling the mouthparts apart with forceps. Hemolymph samples (5 μl) were mixed with 45 μl 50% methanol and stored overnight at −20°C. The samples were vigorously mixed with 150 μl chloroform for 1 min and centrifuged for 10 min at 4°C to separate the aqueous layer. The supernatant was stocked at −20°C or directly diluted for analysis of liquid chromatography–tandem mass spectrometry (LC-MS/MS) (XeVO TQ-MS, Waters) using an ACQUITY UPLC BEH C18 column (1.7 μm×2.1 mm×100 mm).

### Quantitative assay for L-glutamate

The brains of third-instar larvae were dissected and homogenized in PBS. These samples were then used for quantification of L-glutamate via a high-sensitive fluorometric L-glutamate assay kit (STA-674, Cell Biolabs). Following the manufacturer's instructions, 50 μl supernatant of homogenized sample lysates was loaded into 96 wells of a black microplate. Following the addition of 50 μl Reaction Mix (100 μM fluorometric probe, 0.2 U/ml horseradish peroxidase, 0.08 U/ml glutamate oxidase, 0.5 U/ml glutamate-pyruvate transaminase and 200 μM L-alanine) to each sample, the wells were left for 30 min at 37°C. Readout included information for excitation at 550 nm and emission at 595 nm.

### Larval crawling assay

Detection and analysis of crawling behavior in *Drosophila* larvae followed previous descriptions (Brooks et al., 2016). In brief, third-instar larvae were selected and placed upon a 200 mm×115 mm×30 mm agar plate using a paintbrush at 25°C. Each agar plate contained a solution consisting of 1% agar, 0.1 M sucrose and Brilliant Blue dye (in order to provide a dark background for contrast enhancement). The plate was then transferred to a light-, temperature- and humidity-controlled incubator. A camera was placed onto a tripod and focused on the plate. Larvae were then allowed to move for 4 min, during which time they were recorded using a video camera. All genotypes were recorded over approximately the same circadian time period (between 6 h and 9 h after the lights were turned on), although no circadian locomotive rhythms have been reported in larvae (Aleman-Meza et al., 2015). Following video collection, the middle 2 min of each video was converted to .avi and analyzed for crawling movements using the ImageJ (http://imagej.nih.gov/ij/) Plugin wrMTrck (http://www.phage.dk/plugins/wrmtrck.html), which was also used to analyze the speed and length of movement. Related parameters were set as in Brooks et al. (2016). At least three biological repeats were performed for each genotype.

### AO staining

The dissected larval brains were stained with 1.6 μM AO (Sigma-Aldrich) at room temperature (RT) for 5 min. After washing and mounting, samples were imaged using Leica SP5 confocal microscopy.

### Immunostaining and imaging

Dissected larval brains were fixed in 4% paraformaldehyde for 20 min and washed with PBS with 0.5% Triton X-100 (0.5% PBST) three times. The brains were blocked in 2% bovine serum albumin with 0.5% PBST for 30 min at RT. Subsequently, the samples were washed three times with 0.5% PBST. Brain samples were incubated overnight at 4°C in primary antibodies. The following antibodies were used: anti-L-glutamate (1:100; ab9440, Abcam) for L-glutamate staining; anti-GFP (1:200; GTX113617, GeneTex) for the *gstD-GFP* reporter assay; anti-ELAV [1:50; 9F8A9, Developmental Studies Hybridoma Bank (DSHB)] for marking all differentiated neurons; and anti-capase-3 (1:200; 9661, Cell Signaling Technology) for the cell apoptosis assay; as well as anti-Na^+^/K^+^-ATPase (1:200; a5, DSHB). The brains were then washed with 0.5% PBST, followed by application of a secondary antibody (1:400): goat anti-mouse (Abcam) conjugated with Alexa Fluor 488 (A-11001) or goat anti-rabbit conjugated with Alexa Fluor 647 (A-21244). Brains samples were mounted with VECTASHIELD^®^ mounting medium (H-1500, Vector Laboratories) and imaged by Leica SP5 confocal microscopy.

### Lipid peroxidation assay

The brains of third-instar larvae were dissected as above. For immunostaining purposes, lipid peroxidation was exposed by a C11-BODIPY 581/591 fluorescence probe (Bailey et al., 2015). The brains were incubated in Schneider's medium (which was used throughout this protocol) containing 10% fetal bovine serum and 2 μM C11-BODIPY 581/591 (D3861, Invitrogen) for 30 min, followed by washing and mounting with Schneider's medium. The image was immediately captured from the non-oxidized (595 nm) and oxidized (520 nm) spectra by confocal microscopy. For quantification of lipid peroxidation using the malondialdehyde (MDA) assay (Tsikas, 2017), the brain samples were dissected in PBS with 0.05% butylated hydroxytoluene prior to homogenization. In order to determine the concentration of MDA, a TBARS assay kit (OxiSelect™ TBARS Assay Kit-MDA Quantitation, Cell Biolabs) was used, with spectrometric read absorbance at 532 nm. Triplicate experiments were performed.

### Brain histology

Heads of 7-day-old flies were collected using scissors and fixed with Bouin's solution (Polysciences) before being rotated at RT for 5 days. Prior to Hematoxylin and Eosin staining, the head samples were processed by a standard protocol as described previously (Drobysheva et al., 2008): dehydration, clearing, infiltration, embedding and sectioning. Six to seven serial sections of each brain were taken. The number of vacuoles in each section was counted, with the median number of lesions calculated per brain.

### SDS-PAGE and western blotting

Protein lysates were made from larval brains that were collected and homogenized in RIPA lysis buffer containing protease inhibitors and phosphatase inhibitors (Roche). After boiling the lysates, the samples were loaded into a 15% SDS-PAGE gel for electrophoretic separation. For analysis of the western blot, the separated proteins were transferred onto a PVDF membrane and blocked with 5% low-fat milk prior to hybridization with primary anti-actin (anti-JLA20; 1:50; DSHB) or anti-GFP (1:5000; GeneTex) antibody. Secondary antibodies were peroxidase-labeled anti-mouse IgG or anti-rabbit IgG (1:400; Life Technologies). Immunoblot signals were developed by enhanced chemiluminescence (ECL solution, WBKLS0500, Millipore).

### ERG

First, 0-, 7- and 14-day-old flies were fixed in one direction on glass slides using non-toxic glue. Then, 2 M NaCl (for use as a conductive medium) was used to fill both recording and reference electrodes. A reference electrode was placed in the torso of each fly and recording electrodes were put over the retina. Electrode voltage was amplified using a Digidata 1440A, filtered through a Warner IE-210, and the Clampex 10.1 software (Axon Instruments) was used for all recordings. A light stimulus was provided in 1 s pulses via a computer-controlled red LED system (Schott MC1500). All experiments were conducted in duplicate or triplicate with at least ten recordings completed for each genotype and experimental condition.

### PCR and RT-qPCR

For PCR analysis, genomic DNA was extracted from the male *dDBT^Δ^/Y* or *W^1118^* larvae and PCR products were separated in a 1% agarose gel. For RT-qPCR analysis, total RNA from the homogenized third-instar larvae was collected by TRIzol isolation (Invitrogen) as previously described (Rio et al., 2010). From the total RNA, cDNA was reverse-transcribed using the SuperScript III kit (Invitrogen) and mRNA expression levels were determined using SYBR^®^Green-based RT-qPCR. An AB ViiA-7 Real-Time PCR system was used to detect Ct values. Data were analyzed using QuantStudio™ software. The relative mRNA expression levels were normalized to *rp49* (also known as *RpL32*)*.* Three biological repeats were conducted for each genotype.

### Primers

For determining genomic mutation by general PCR analysis, the following primers were used: *dDBT* forward 5′-AAGGCATCGGTGACCATCAC-3′, *dDBT* reverse 5′-AGCCTTGATGCAGAACGGC-3′; Up forward 5′-TTTCCATCTAGCGTCCGATT-3′, Up reverse 5′-CGAGGGTTCGAAATCGATAA-3′; Down forward 5′-AACGCAAGCAAATGTGTCAG-3′, Down reverse 5′-GAAACGAAGCGATACGAAGG-3′.

For determining mRNA expression by RT-qPCR analysis, the following primers were used: *DptA* forward 5′-GTTCACCATTGCCGTCGCCTTAC-3′, *DptA* reverse 5′-CCCAAGTGCTGTCCATATCCTCC-3; *CecA* (also known as *CecA1*) forward 5′-ATGAACTTCTACAACATCTTCGT-3′, *CecA* reverse 5′-ATTGTGGCATCCCGAG-3′; *AttA* forward 5′-GTGGTGGGTCAGGTTTTCGC-3′, *AttA* reverse 5′-TGTCCGTTGATGTGGGAGTA-3′; *rp49* forward 5′-AGATCGTGAAGAAGCGCACCAAG-3′, *rp49* reverse 5′-CACCAGGAACTTCTTGAATCCGG-3′; *dDBT* forward 5′-AGATCGTGAAGAAGCGCACCAAG-3′, *dDBT* reverse 5′-TTGCATCAACAGGGTCATGT-3′.

### Metformin administration

Metformin (D150959, Sigma-Aldrich) was prepared to concentrations of 2, 10 and 100 mM, and added to *Drosophila* regular food. For Metformin exposure experiments, first-instar larvae were picked and reared in Metformin-containing food continuously. Apart from the eclosion assays, third-instar larvae were used for all related experiments after 10 mM Metformin administration.

### Lifespan assay

One hundred adult female *W^1118^* or *dDBT^Δ^/+* flies were divided evenly into five vials and housed at 25°C. Flies were transferred to new vials every day without anesthesia. After vial replacement, dead flies were recorded.

### Statistical analysis

Results were analyzed and presented as bar graphs using GraphPad Prism 6.0. For the eclosion rate experiments, post hoc pairwise chi-squared tests utilizing a Bonferroni correction were computed in R to compare wild-type eclosion rates with individual mutant eclosion rates. Kaplan–Meier survival curves were created for visualizing the lifespan data, and the log-rank (Mantel–Cox) test was used for life span analyses. For other experiments, unpaired Student's *t*-tests were used for statistical analysis. Significance was defined as *P*<0.05, and *P*<0.01 was defined as indicating a trend. All tests used a (corrected) significance level of *P*<0.01.

## Supplementary Material

Supplementary information
