# Supplementary material for: Loss of the Drosophila branched-chain α-ketoacid dehydrogenase complex results in neuronal dysfunction
Source: Dis Model Mech. 2020 Aug 27;13(8):dmm044750. doi: 10.1242/dmm.044750 (PMC7473638; doi:10.1242/dmm.044750)
Supplement: Supplementary information [file dmm-13-044750-s1.pdf]

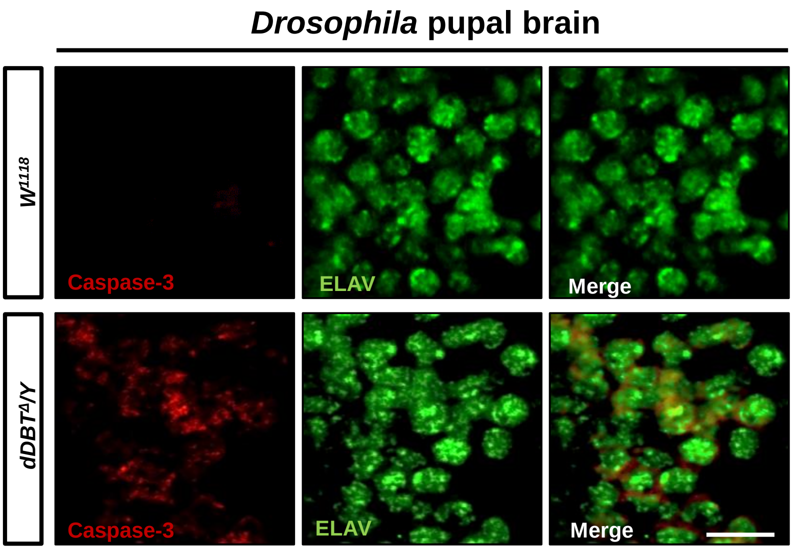

Fig. S1. The *dDBT* deficiency induces apoptosis in the pupal brain. Apoptosis assay of pupal brains co-stained with anti-ELAV and anti-Caspase-3 antibodies. The presented images of central brain regions of *Drosophila* pupae were captured using fluorescence confocal microscopy with a 63x oil objective and 5-fold magnification. Scale bar: 10 µm.

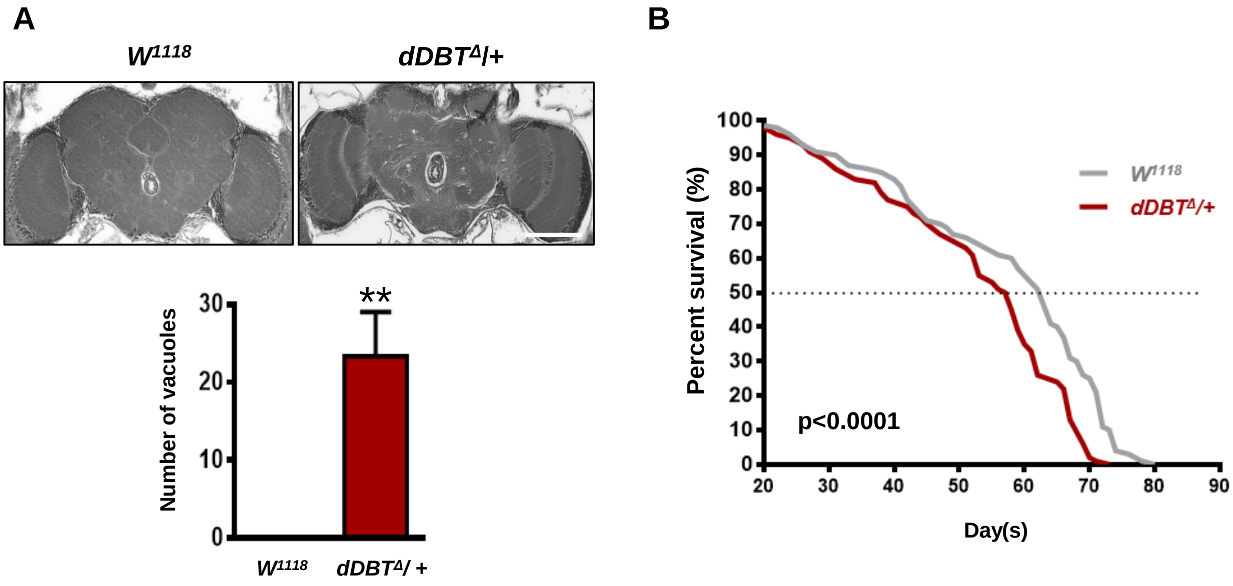

Fig. S2. Heterozygous *dDBT* mutant exhibits brain damage and a shortened lifespan. (A) Histological analysis of brains section of 7-day-old *Drosophila* from w^{1118} or heterozygous *dDBT* mutant lines and the quantification of the number of vacuoles in each brain. Three brains were used for each group/experiment. Three biological repeats were conducted, and *p* values were calculated using a Student's t-test. $*p < 0.05$; $**p < 0.01$; Error bars indicate standard deviation. (B) Lifespan evaluation of the heterozygous *dDBT* mutants compared to wild type W^{1118} flies (total n=100/each group), which was analyzed by Log-rank(Mantel-Cox) test analysis. Scale bar: 100 µm.

Disease Models & Mechanisms • Supplementary information

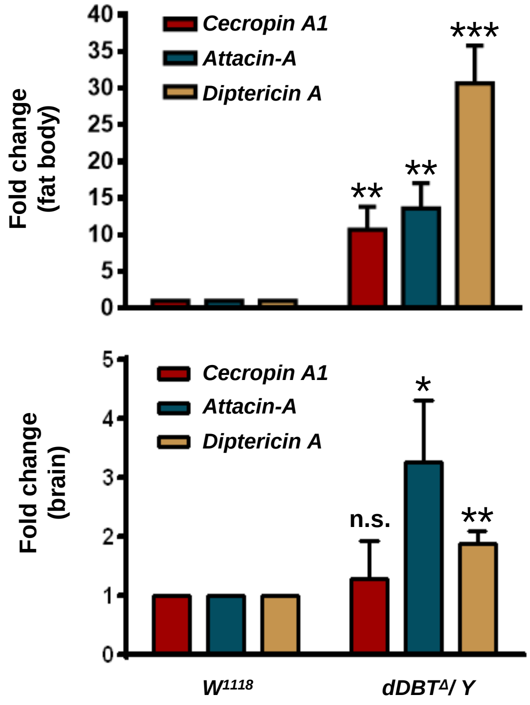

Fig. S3. A higher systemic immune response is induced by the loss of *dDBT*. Quantitative RT-PCR analysis of antimicrobial peptide mRNA expression in the tissues of fat bodies or brains from w^{1118} or *dDBT* mutants. Thirty brains were used for each group/experiment. Three biological repeats were conducted, and *p* values were calculated using a Student's t-test. *$*p < 0.05$*; *$**p < 0.01$*; *** *$p<0.001$.* n.s., not statistically significant; Error bars indicate standard deviation.
